# Infections caused by mycobacterium tuberculosis in patients with hematological disorders and in recipients of hematopoietic stem cell transplant, a twelve year retrospective study

**DOI:** 10.1186/1476-0711-6-16

**Published:** 2007-11-16

**Authors:** Khalid Ahmed Al-Anazi, Asma Marzouq Al-Jasser, David Alan Price Evans

**Affiliations:** 1 Section of Adult Hematology and Hematopoietic Stem Cell Transplant, King Faisal Cancer Centre, King Faisal Specialist Hospital and Research Centre, P. O. Box: 3354, Riyadh-11211, Saudi Arabia; 2 Department of Pathology, Armed Forces Hospital, P.O. Box: 7897, Riyadh-11159, Saudi Arabia; 3 Department of Medicine, Armed Forces Hospital, P.O. Box: 7897, Riyadh-11159, Saudi Arabia

## Abstract

**Background:**

Tuberculous infections in patients with hematological disorders and hematopoietic stem cell transplant vary in incidence, complications and response to treatment.

**Methods and materials:**

A retrospective study of patients with various benign and malignant hematological disorders and recipients of hematopoietic stem cell transplant who were treated at Riyadh Armed Forces Hospital, Saudi Arabia between January 1991 and December 2002 and who developed tuberculous infections was conducted.

**Results:**

Tuberculous infections occurred in eighteen patients with hematological disorders and hematopoietic stem cell transplant. The main associated factors were: reduced immunity due to the primary hematological disorder, age more than 50 years and the administration of cytotoxic chemotherapy, steroids or radiotherapy. These infections frequently involved the lungs and predominantly occurred in males and in patients with chronic myeloproliferative disorders, myelodysplastic syndrome and acute myeloid leukemia. In patients treated with intravenous cytotoxic chemotherapy, tuberculous infections tended to occur earlier and also tended to be more disseminated compared to infections occurring in patients treated with oral chemotherapy. Anti-tuberculous treatment was given to 16 patients and it was successful in 15 of these patients.

**Conclusion:**

Tuberculous infections cause significant morbidity and mortality in patients with various hematological disorders and in recipients of hematopoietic stem cell transplant. The early administration of anti-tuberculous therapy and compliance with drug treatment are associated with successful outcomes while delayed management, drug resistance and the presence of miliary infections are associated with poor prognosis and high mortality rates.

## Introduction

Tuberculous infections (TIs) are serious and life-threatening complications in patients with malignant hematological disorders and in recipients of hematopoietic stem cell transplant (HSCT) [1-7]. Reactivation of latent tuberculosis (TB) may be induced by the administration of cytotoxic chemotherapy and high dose corticosteroid therapy [1,3,4,8]. A high index of suspicion should be maintained in immunocompromised individuals presenting with clinical and radiological manifestations compatible with TIs [6,7,9-16]. At times, an empirical anti-tuberculous therapy should be employed to prevent further clinical deterioration and a possible lethal outcome that may be associated with delayed diagnosis and late institution of treatment [17].

The efficacy of anti-TB chemotherapy in patients with hematological malignancies and in recipients of HSCT is well documented [4,9]. However, drug resistance, non-compliance with medications and the presence of advanced and miliary forms of tuberculosis remain real challenges [4,18,19].

This retrospective analysis of TIs in immunocompromised hosts living in endemic areas was performed to explore the incidence, the complications of these infections and their response to treatment.

## Methods and materials

Riyadh Armed Forces Hospital is a tertiary care center comprising 1200 beds with specialty services including: intensive care, hematology/oncology as well as solid organ and HSCT. A retrospective study was conducted between January 1991 and December 2002. The medical records and the microbiological data of patients with benign and malignant hematological disorders and recipients of HSCT who developed TIs were reviewed.

### Mantoux test

Injection of 0.1 ml of tuberculin solution intradermally. The test is read at 48 to 72 hours. A positive result is indicated by an induration of at least 10 mm in diameter.

## Results

During the study period (12 years), 18 patients developed TIs. Twelve were males and 6 were females with a male : female ratio of 2:1. The median age of the patients was 48.5 years (range 19 to 75 years). The predominant primary hematological disorders were: chronic myeloproliferative disorders (6 patients), acute myeloid leukemia (AML) (4 patients) and myelodysplastic syndrome (MDS) (3 patients) (Table 1). During the study period, TIs developed in: 4 out of 128 patients with AML treated at our institution (incidence: 3.1%); 3 out of 51 patients with chronic myeloid leukemia (CML) (5.9%); 3 out of 41 patients with MDS (7.3%); 1 out of 25 patients with severe aplastic anemia (SAA) (4%); 1 out of 10 patients with hairy cell leukemia (HCL) (10%); 1 out of 12 patients with essential thrombocythemia (8.3%); 1 out of 13 patients with polycythemia rubra vera (7.7%); 1 out of 14 patients with multiple myeloma (7.1%); 1 out of 16 patients with Hodgkin's lymphoma (6.3%) and 1 out of 36 patients with thrombocytopenic purpura (2.8%). No single TI was encountered in any of the 109 patients with acute lymphoblastic leukemia (ALL) or in the 22 patients with chronic lymphocytic leukemia (CLL) treated at our institution during the study period (Table 1). The main associated factors for the development of TIs were: reduced immunity due to the primary hematological disease, age more than 50 years and the administration of cytotoxic chemotherapy or steroid treatment (Table 2).

**Table 1:** Details of the study patients

Number	Age (years)	Sex	Primary hematological disorder	Organs involved by tuberculosis	Diagnosis of tuberculosis	Treatment/Outcome of tuberculosis
1	55	Male	Chronic myeloid leukemia	Intestine, mesentry and abdominal lymph nodes	Positive lymph node histology and cultures	Given/Successful
2	40	Male	Acute myeloid leukemia	Lungs	Positive sputum cultures	Given/Successful
3	19	Female	Acute myeloid leukemia	Lungs	Positive sputum cultures	Given/Successful
4	20	Female	Hodgkin's lymphoma	Lungs, liver and abdominal lymph nodes	Positive sputum cultures	Given/Successful
5	33	Female	Thrombocytopenic purpura	Cervical lymph nodes	Positive lymph node cultures	Given/Successful
6	70	Male	Hairy cell leukemia	Lungs, liver, spleen, cerebrospinal fluid and bone marrow	Positive cultures(4 sites) and bone marrow histology	Given/Unsuccessful *Multi-drug resistance
7	32	Male	Multiple Myeloma	Lungs	Positive sputum cultures	Given/Successful
8	36	Female	Essential thrombocythemia	Lungs	Positive sputum cultures	Given/Successful
9	24	Male	Acute myeloid leukemia	Lungs, bone marrow and liver.	Positive bronchial lavage	Not given/Death
10	45	Male	Severe aplastic anemia	Lungs	Positive sputum cultures	Not given/Lost follow up
11	70	Male	Myelodysplastic syndrome	Lungs and cervical lymph nodes	Empirical/positive lymph node histology	Given/Successful
12	62	Male	Polycythemia rubra vera	Cervical lymph nodes	Empirical/positive lymph node histology	Given/Successful
13	38	Male	Acute myeloid leukemia	Lungs, bone marrow, psoas muscle and vertebrae.	Empirical/positive muscle and bone marrow histology	Given/Successful
14	52	Female	Chronic myeloid leukemia	Lungs	Empirical/positive serology	Given/Successful
15	59	Male	Myelodysplastic syndrome	Lungs	Empirical/positive serology	Given/Successful
16	75	Male	Myelodysplastic syndrome	Cervical spine	Empirical	Given/Successful
17	60	Female	Myelofibrosis	Lungs	Empirical	Given/Successful
18	65	Male	Chronic myeloid leukemia	Lungs	Empirical	Given/Successful

**Table 2:** The associated factors for the development of tuberculosis

**Predisposing factor**	**Number of patients**	**Percentage of patients**
Age more than 50 years	9	50%
Cytotoxic chemotherapy, intravenous and oral	11	61.1%
Steroid therapy	5	27.8%
Radiotherapy	3	16.7%
Hematopoietic stem cell transplant	3	16.7%
Graft versus host disease	3	16.7%
Other medical illnesses causing reduced immunity	2	11.1%

Isolates from 10 patients were identified as *M. tuberculosis*. The *M. tuberculosis *isolates were sensitive to the first line drugs [rifampicin, isoniazid (INH), pyrizinamide and ethambutol] except one isolate which was multidrug resistant. The diagnoses of TIs were confirmed in the 10 patients who had positive cultures. In the remaining 8 patients, the diagnoses of TIs were made on an empirical basis ie having clinical (weight loss, fever, malaise, sweating, chronic cough etc) and radiological (pulmonary fibrosis, cavitation, calcification or nodular shadows) manifestations strongly suggestive of TIs. Three of these patients had histological changes (granuloma formation with or without caseation necrosis on biopsies taken from lymph nodes, bone marrow or muscle) compatible with TB and 2 other patients had positive TB serology (high IgM antibody levels). All the 8 patients in whom the diagnoses of TIs were not confirmed by positive cultures had positive mantoux tests and all of them had clinical responses to the anti-TB chemotherapy. Those findings strongly supported the diagnoses of TIs in these 8 patients (Table 1).

Anti-TB treatment was given to 16 patients. In 2 patients, no anti-TB chemotherapy was administered as in one of these patients the diagnosis of TB was established after his death and the other patient unfortunately lost follow up at our institution. Out of the 16 treated patients, 15 patients were successfully treated and 13 of them were compliant with the treatment (Table 1). In one patient, anti-TB chemotherapy failed to control the infection due to having a multidrug resistant organism. In the patients who had pulmonary or disseminated TIs, 4 anti-TB drugs were employed and the treatment was given for a total duration of 12 months. However, in patients having localized disease eg lymphadenopathy: 3 anti-TB drugs were given for a total duration of 9 months. Anti-TB chemotherapy was given according to the sensitivity testing in the patients who had positive TB cultures. In the patient who had drug resistance, the treatment was modified according to sensitivity results. INH chemoprophylaxis was successful in preventing the reactivation of TIs in 5 patients, 2 of them had HSCT done. These 5 patients had developed TIs after establishing the diagnoses of their hematological disorders and they had been treated with anti-tuberculous chemotherapy, then they were given INH chemoprophylaxis to prevent the reactivation of their old TIs as they susequently received further courses of cytotoxic chemotherapy and/or HSCT in order to control/cure their hematological disorders. Forteen patients presented with pyrexial illnesses while 4 patients had no pyrexia at presentation. No cardiac or adrenal involvement was reported in any of the patients studied.

Eleven patients had received steroids or cytotoxic chemotherapy to treat their primary hematological disorders. In the 6 patients who had received IV cytotoxic chemotherapy (idarubicin, cytosine arabinoside, cyclophosphamide): TIs occurred at a median time of 3 months after the last course of chemotherapy given and at a median time of 11.5 months from the date of the diagnosis of the primary hematological disorder. In the 5 patients who had received oral chemotherapy group (hydroxyurea and steroids); TIs occurred at a median time of 3 months after the last course of chemotherapy given and at a median time of 35 months from the date of the diagnosis of the primary hematological disorder. Disseminated TB infections were encountered in three patients (50%) in the IV chemotherapy group of patients and only in one patient (20%) belonging to the oral chemotherapy group.

The diagnoses of TIs were made after the diagnoses of the primary hematological disorder in 12 patients, both TIs and the primary hematological disorder were diagnosed simultaneously in 5 patients while in 1 patient the TI was diagnosed when the patient presented with anemia and monoclonal gammopathy 2 years before the diagnosis of the primary hematological disorder (multiple myeloma). In the subgroup of patients in whom TIs were diagnosed after the diagnoses of the primary hematological disease, the diagnoses of TIs were made at a median time of 15 months after the diagnoses of the primary hematological disorder.

The blood indices of the study patients at the presentation of the TIs were as follows: pancytopenia (leucopenia: white cell count < 4 × 10^9^/L, anemia: hemoglubin < 11 g/dL and thrombocytopenia: platelet count < 150 × 10^9^/L) was seen in 5 patients (27.8%); normal, anemia or mixed blood picture in 4 patients each (22.2%) and thrombocytopenia in 1 patient (5.6%). The chest radiographic appearances at the presentation of TIs were as follows: areas of pulmonary consolidation consistent with pneumonia were seen in 10 patients (55.6%); fine reiculonodular shadows consistent with pulmonary fibrosis in 7 patients (38.9%); calcification, pleural effusions or lymph node enlargement in 3 patients each (16.7%) and basal atelectasis, cavity formation or miliary shadows in 1 patient each (5.6%). However, the chest radiographs were found to be normal in 3 patients (16.7%).

TIs occurred in 5 out of 103 patients with a variety of benign and malignant hematological disorders who received HSCT at our institution during the study period. In 2 of these patients, TIs occurred before the HSCT and in the other 3 patients, TIs occurred after the HSCT. The incidence of TIs in HSCT patients at our institution was 2.9%. All the three patients who developed TIs after receiving HSCT had AML, all received IV cytotoxic chemotherapy (cytosine arabinoside and idarubicin), all were given total body irradiation and cyclophosphamide conditioning therapy prior to the HSCT, all developed graft versus host disease (GVHD) treated with steroid therapy and all had pulmonary involvement (Table 3). Two HSCT patients had primary TIs. In both patients; the initial clinical, radiological and microbiological screenings for TB at the presentations of their primary hematological disorders were negative. One other patient had reactivation of an old TB infection one year following a successful HSCT. This patient had pulmonary TB diagnosed at the time of the diagnosis of her AML. After treating both the AML and the pulmonary TB, the patient had a successful allograft that was complicated by an acute then a chronic GVHD that were treated with steroids. Two of the HSCT patients had positive TB cultures and in the third patient, TI was diagnosed empirically. Anti-TB chemotherapy was successful in the 2 patients who received it. It was noticable that none of the 52 allogeneic HSCT recipients having primary hematological disorders other than AML ie patients having disorders such as SAA, MDS, ALL developed TIs. It was also noted that TIs were not encountered in recipients of other forms of HSCT eg autologous grafts (21 patients).

**Table 3:** Tuberculosis in HSCT patients

Number	Primary hematological disorder	Number of CCC prior to HSCT	HSCT conditioning regimen used	GVHD Type/Organs involved	Treatment of GVHD	Organs involved by TI	Time interval between HSCT and TI
1	AML, M2	3	TBI + CPM	Acute/Liver	Steroids	Lungs, liver and bone marrow	1 month
2	AML, M3	4	TBI + CPM	Acute/Skin	Steroids	Lungs	5 months
3	AML, M4	4	TBI + CPM	Acute/Skin	Steroids	Lungs	12 months

GVHD: Graft versus host disease

TBI: Total body irradiation

TI: Tuberculous infection

HSCT: Hematopoietic stem cell transplant

CPM: Cyclophosphamide

CCC: Courses of cytotoxic chemotherapy

AML: Acute myeloid leukemia

M2, M3, M4: Subtypes of AML

## Discussion

Infection is a serious complication in patients with malignant hematological disorders [15]. The prevalence of TB in patients with hematological malignancies ranges between 2.1 and 2.6% [17]. Clinically evident TB can antedate malignancy, both may present simultaneously or TB may develop after the treatment of the malignant disorders [3,8]. The average time between the completion of cytotoxic chemotherapy and the development of TB is 18 to 20 months [3,4]. Persistent pyrexia in patients with malignant hematological disorders, in remission, may serve as a diagnostic marker for TB [14]. Difficulties in establishing the diagnosis of TB can be partially explained by the limitations of most of the existing diagnostic methods [17]. The bacteriological, the histological and the new molecular diagnostic techniques should be employed in patients suspected to have TB [15,17].

There is an association between TB and HCL [5]. In such patients, TB is characterized by the presence of few pulmonary symptoms, scarce microbiological findings and a miliary distribution [5]. In patients with chronic myeloproliferative disorders, the incidence of TB is high and the infection is usually disseminated [2]. In the patients studied, the incidence of TIs was relatively high in those having chronic myeloproliferative disorders, MDS, AML and SAA. In the patients who received steroids or cytotoxic chemotherapy, TIs occurred at a median time of 3 months from the last course of therapy given. In the 6 patients who had chronic myeloproliferative disorders, disseminated TI was found in 1 patient only and positive cultures and histology were encountered in 3 of these patients.

The increased utilization of anti-neoplastic agents in the treatment of acute leukemia is associated with an increase in the incidence of opportunistic infections, eg *M. tuberculosis*, that may be complicated by systemic dissemination and multiorgan failure [1]. TB should also be considered as a possible cause of hepatosplenic abscesses during the prolonged periods of neutropenia following the courses of cytotoxic chemotherapy given for patients with acute leukemia in the areas that are endemic for TB [20]. Once anti-TB treatment is instituted, a successful outcome can be achieved in up to 90% of patients although a lethal outcome has been reported in patients with miliary TB [4]. All of our 4 patients with acute leukemia who developed TIs had AML and all had pulmonary involvement as well as positive cultures or histology.

TB is a serious and a life threatening infectious complication which may cause fatal sepsis and a rapidly progressive illness in recipients of HSCT [6,7]. TB is relatively rare (incidence 0.6–2.7%) in HSCT patients despite their severely immunocompromised state [6,7,10-13,21-23]. TB occurs mainly in recipients of T-cell depleted allogeneic grafts and in patients developing GVHD [21]. The slow growth of the organism can lead to a late diagnosis and a delay in the institution of treatment [10]. A high index of suspicion as well as pre and post-transplant follow up are required in countries where TB is common [6,10,22]. TB should also be considered in the differential diagnosis of unexplained infections in recipients of HSCT [7,11]. Pre-transplant chest radiographic abnormalities suggesting TB should be carefully evaluated [12]. GVHD significantly increases the morbidity and the mortality related to the pulmonary complications in HSCT recipients [23]. Rapidly progressive TIs and miliary forms of TB should be considered in recipients of HSCT presenting with an unexplained pyrexia and diffuse reticulonodular shadows [6,13]. The following factors are associated with high mortality rates in patients with miliary TB: delayed diagnosis, late administration of treatment and the presence of complications such as disseminated intravascular coagulation (DIC), septic shock, adult respiratory distress syndrome and multiorgan failure [19]. All our HSCT patients who developed TI had: AML, several courses of intravenous cytotoxic chemotherapy with idarubicin and cytosine arabinoside and allogeneic grafts. They all had total body irradiation and cyclophosphamide conditioning and acute GVHD treated with steroids. In 1 of these patients, TB presented acutely and was miliary in distribution causing a multisystem failure.

In patients with pulmonary TB: anemia, leucocytosis, thrombocytosis, thrombocytopenia and a high erythrocyte sedimentation rate (ESR) can occur [24]. Miliary TB can present with pancytopenia, bone marrow necrosis and granuloma formation [25,26]. TB may present with DIC and should be considered in the differential diagnosis of thrombocytopenia particularly in patients having abnormal chest radiographs [13,19,27]. In immunocompromised patients, TB may coexist with fungal infections and may be localized or disseminated [19,28]. Even in apparently healthy individuals living in endemic areas, TB is the commonest infectious cause of pyrexia of unknown origin [29]. TB was the cause of thrombocytopenia in 1 of our patients. Also, TB involved the psoas muscle and it co-existed with a disseminated aspergillus infection in another patient.

INH prophylaxis should be considered in HSCT recipients with: healed TB receiving corticosteroid therapy, known family contacts, previous bacillus Calmette-Guerin (BCG) immunotherapy or history of an inadequately treated TB [8,12]. INH is effective in the prevention of active TB in patients with malignant hematological disorders and is well tolerated [30]. Anti-TB drugs are associated with several adverse effects that include: fever, leucopenia, agranulocytosis, allergic reactions and an elevation of liver enzymes [31,32]. Fortunately, most of these toxic reactions are reversible [31]. In countries where drug resistance is high, three or four anti-TB drugs should be employed [16]. Recurrence of TB, increasing multiple resistance to the current drugs and non-compliance with treatment are challenging problems [18]. INH prophylaxis was used successfully in 5 of our patients. No major toxicity related to anti-TB therapy was encountered in our group of patients. Two of our patients were non-compliant with treatment and 1 patient had a multi-drug resistant strain.

We acknowledge that the number of the patients studied was rather small and that retrospective analyses have their own limitations. Therefore, further multicentric prospective studies are required to explore the various aspects of TIs in immunocompromized individuals living in areas that are endemic for TB.

In conclusion: TB is a serious infection in immunocompromised individuals. Sometimes, empirical anti-TB therapy is necessary when the clinical and the radiological features are strongly suggestive of TI particularly in patients living in endemic areas. In our patients, TIs occurred predominantly in males and in patients with chronic myeloproliferative disorders, myelodysplasia and AML. The more intensive the cytotoxic chemotherapy (repeated courses of intravenous chemotherapy rather than oral chemotherapeutic agents) given for the treatment of the primary hematological disorder, the earlier the development of TI and the more likely that the infection would be disseminated. In HSCT patients, TIs occurred in patients with AML, those treated with repeated courses of intravenous cytotoxic chemotherapy, those who received allogeneic grafts and conditioned with total body irradiation and cyclophosphamide and in patients having GVHD treated with corticosteroids. The absence of TIs in patients with ALL and CLL and the predominant lung involvement were striking features in our group of patients.

## Competing interests

The author(s) declare that they have no competing interests.

## Authors' contributions

All the authors participated in the management of the patients presented. They all participated fully at various stages of the study. All the authors read and approved the final form of the manuscript.
